# MiRNA-101 inhibits breast cancer growth and metastasis by targeting CX chemokine receptor 7

**DOI:** 10.18632/oncotarget.5067

**Published:** 2015-09-01

**Authors:** Jun-Tang Li, Lin-Tao Jia, Ning-Ning Liu, Xiao-Shan Zhu, Qin-Qin Liu, Xiu-Li Wang, Feng Yu, Yan-Li Liu, An-Gang Yang, Chun-Fang Gao

**Affiliations:** ^1^ Centre of Inflammation and Cancer Research, 150th Central Hospital of PLA, Luoyang, Henan 471031, China; ^2^ State Key Laboratory of Cancer Biology, Department of Immunology, Fourth Military Medical University, Xi'an, Shaanxi 710032, China; ^3^ State Key Laboratory of Cancer Biology, Department of Biochemistry and Molecular Biology, Fourth Military Medical University, Xi'an, Shaanxi 710032, China

**Keywords:** breast cancer, MiR-101, CXCR7, proliferation, metastasis

## Abstract

Whereas miR-101 is involved in the development and progression of breast cancer, the underlying molecular mechanisms remain to be elucidated. Here, we report that miR-101 expression is inversely correlated with the clinical stage, lymph node metastasis and prognosis in breast cancers. Introduction of miR-101 inhibited breast cancer cell proliferation and invasion *in vitro* and suppressed tumor growth and lung metastasis of *in vivo*. CX chemokine receptor 7 (CXCR7) is a direct target of miR-101, positively correlating with the histological grade and the incidence of lymph node metastasis in breast cancer patients. The effects of miR-101 were mimicked and counteracted by CXCR7 depletion and overexpression, respectively. STAT3 signaling downstream of CXCR7 is involved in miR-101 regulation of breast cancer cell behaviors. These findings have implications for the potential application of miR-101 in breast cancer treatment.

## INTRODUCTION

Breast cancer (BrC) is a common and highly lethal malignancy [1]; its rapid recurrence and poor survival rates are caused by sustained proliferation, activated invasion and metastasis, and resistance to cell death. Therefore, identification of molecules that promote apoptosis and suppress the proliferation and metastasis of BrC cells may provide novel targets for clinical therapies.

MicroRNAs (miRNAs) are a class of highly-conserved small RNAs that regulate diverse cellular processes by base-pairing with the 3′-untranslated regions (UTRs) of target mRNAs [2]. Increasing evidence suggests that miRNA dysfunction is involved in BrC development and progression [3, 4]. MiR-101 is frequently downregulated in multiple cancer types, including BrC [5–8]. MiR-101 inhibits BrC cell proliferation and invasion by targeting Stathmin1 and Enhancer of Zeste Homolog 2 (EZH2), and promotes apoptosis by downregulating the expression of Myeloid Cell Leukemia-1 (Mcl-1) and EZH2 [7–9]. Nevertheless, the precise mechanism by which miR-101 inhibits the malignant phenotype of BrC cells remains unknown.

CX chemokine receptor 7 (CXCR7) is involved in various biological processes, such as cell survival, adhesion, and mobility [10]. Overexpression of CXCR7 has been observed in various tumors, including BrC, lung cancer, prostate cancer, glioma, and hepatocellular carcinoma [11–14]. Highly expressed CXCR7 has anti-apoptotic effects in human glioma cells [15]. Miao *et al*. [11] showed that CXCR7 promotes tumor growth in a mouse model of lung cancer and BrC, and that its expression level influences lung metastasis. CXCR7 expression is also correlated with lymph node metastasis and poor prognosis in BrC [16]. Enforced expression of CXCR7 *in vitro* enhances the proliferation of BrC cells [17]; however, in a previous study examining CXCR7-mediated effects on breast tumor growth and metastasis, overexpression of CXCR7 inhibited invasion and metastasis but enhanced primary tumor growth [18]. While the discrepancies in these results could be due to the different cell types, experimental conditions, and/or model systems utilized, the function and regulatory mechanism of CXCR7 in BrC growth and metastasis require further clarification.

Here, we investigated the potential function of miR-101 in BrC carcinogenesis and found that downregulation of miR-101 in BrC tissues was positively associated with advanced clinical stages and metastasis of BrCs, and prognosis of patients. Dual-luciferase reporter assays showed that CXCR7 was targeted by miR-101 directly. In addition, *in vitro* and *in vivo* assays revealed that restoration of miR-101 expression inhibited BrC growth, metastasis, and apoptosis evasion significantly, and these effects were phenocopied and abrogated by silencing and overexpression of CXCR7, respectively. Analyses of the molecular mechanisms involved in these processes revealed that miR-101 reduced BrC tumorigenesis and progression by inhibiting the CXCR7–signal transducer and activator of transcription 3 (STAT3) signaling pathway. We also provide evidence that CXCR7 expression is positively correlated with the histological grade and lymph node metastasis in BrC, whereas these outcomes are inversely correlated with the miR-101 level. Overall, the results presented here elucidate the underlying mechanism by which miR-101 inhibits BrC growth and metastasis.

## RESULTS

### Downregulation of miR-101 is positively correlated with the advanced histological grade, metastasis, and poor prognosis of BrC

**Figure 1:**
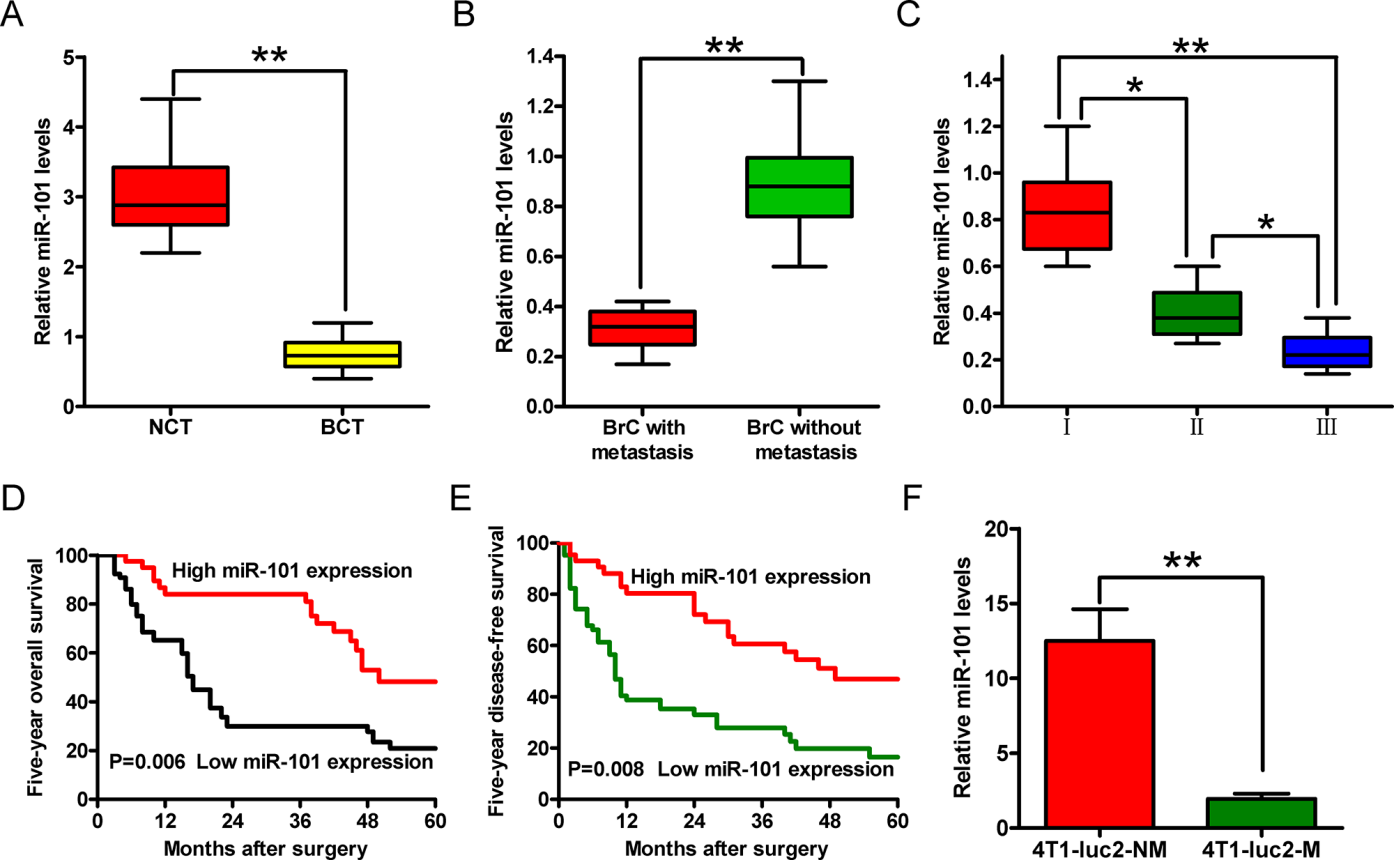
Expression levels of miR-101 in BrC tissues and cell sublines, and their correlations with clinical features **A–C.** Comparisons of the expression levels of miR-101 in BrC tissues (BCT, *n* = 64) and corresponding non-cancerous tissues (NCT, *n* = 64) (A), BrC tissues with (*n* = 48) or without (*n* = 35) lymphatic metastasis (B), and BrC tissues from patients at different clinical stages (WHO I, *n* = 9; WHO II, *n* = 32; and WHO III, *n* = 43) (C) **D, E.** The 5 year overall survival (D) and disease-free survival (E) rates of BrC patients with high (*n* = 44) and low (*n* = 67) levels of miR-101 expression. The patients were divided into the two groups on the basis of the qRT-PCR data. The results were plotted after a review of in-patient records and a follow-up study. The *P*-values were determined using a log-rank test. **F.** The expression levels of miR-101 in BrC cell lines with different metastatic potentials. (A–C, F) The expression level of miR-101 was determined using qRT-PCR and was normalized to that of an endogenous control (U6 RNA). All data are represented as the mean ± SD of three replicates. *P < 0.05 and **P < 0.01.

The miR-101 expression level was determined using quantitative reverse transcription-polymerase chain reaction (qRT-PCR) analyses and was normalized to that of an endogenous control (U6 RNA). The expression level of miR-101 was significantly lower in human BrC tissues than adjacent non-cancerous breast tissues (Figure 1A). Furthermore, miR-101 expression was lower in metastatic than non-metastatic BrC tissues (Figure 1B), and a high expression level was inversely correlated with the histological grade of the tumor (Figure 1C). In addition, the 5 year overall survival and disease-free survival rates of patients with high miR-101 levels were higher than those with low miR-101 levels (Figures 1D and 1E). These results indicate that downregulation of miR-101 expression is positively correlated with the advanced histological grade, metastasis, and poor prognosis of BrC.

To further examine the association between miR-101 and BrC malignancy, we analyzed the miR-101 levels in BrC sublines with different metastatic potentials, namely, 4T1-luc2-M (metastatic) and 4T1-luc2-NM (non-metastatic) cells (Supplementary Figure S1). The miR-101 expression level was lower in the 4T1-luc2-M cell line than the 4T1-luc2-NM cell line (Figure 1F). These results confirmed that reduced miR-101 expression is positively correlated with BrC cell metastasis.

### MiR-101 inhibits the proliferation, apoptosis evasion, migration, and invasion of BrC cells

To explore the biological significance of miR-101 to BrC further, we transfected miR-101 mimics and anti-miR-101 (as-miR-101) into 4T1-luc2-M and 4T1-luc2-NM cells, respectively. As expected, qRT-PCR analyses confirmed that miR-101 level was increased significantly in 4T1-luc2-M cells transfected with the miR-101 mimic, and decreased significantly in 4T1-luc2-NM cells transfected with as-miR-101 (Supplementary Figure S2).

**Figure 2:**
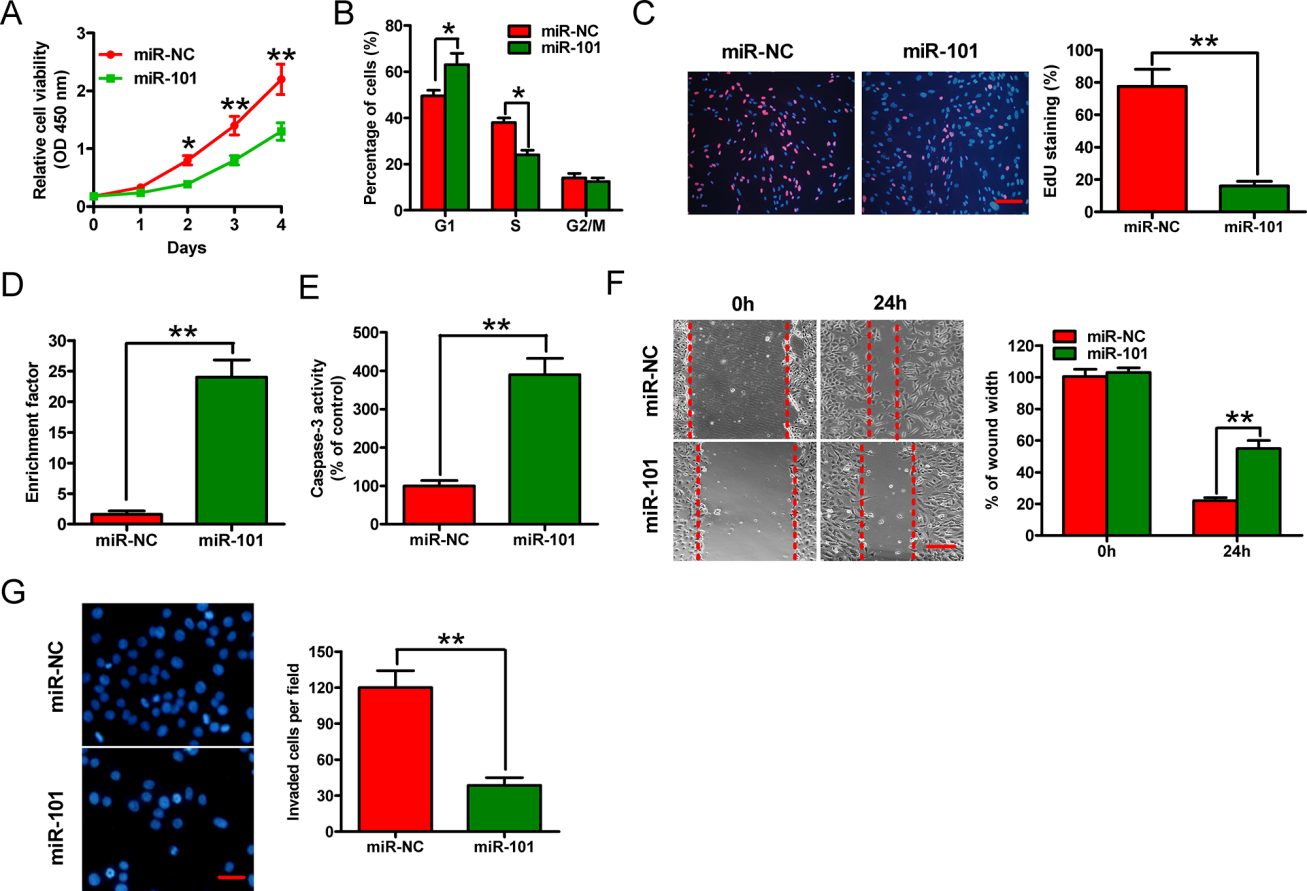
Effects of miR-101 on *in vitro* proliferation, apoptosis, migration, and invasion of BrC cells The 4T1-luc2-M cell line was transfected with 100 nM miR-101 mimic or the negative control miR-NC mimic. **A.** CCK-8 assay of cells transfected with miR-101 or miR-NC for the indicated number of days. **B.** Fluorescence-activated cell sorting assay of cells transfected with miR-101 or miR-NC, showing the effects on cell cycle progression. **C.** EdU incorporation assay of cells transfected with miR-101 or miR-NC. Scale bar = 20 μm. **D, E.** Nucleosomal fragmentation (D) and caspase-3 activity (E) assays of cells transfected with miR-101 or miR-NC. **F, G.** Wound-healing (F) and transwell (G) assays of cells transfected with miR-101 or miR-NC showing the effects on migration and invasion of 4T1-luc2-M cells. Scale bars = 10 μm (migration) or 20 μm (invasion). (C, F, G) Representative images are shown. (A–G) Data are represented as the mean ± SD of three replicates. *P < 0.05 and **P < 0.01.

Transfection of 4T1-luc2-M cells with the miR-101 mimic for 2, 3, or 4 days inhibited the viability of the cells (Figure 2A). The proportion of miR-101-transfected cells at the G0/G1 stage (64%) was higher than the proportion of control cells at this stage (47%) (Figure 2B), indicating that miR-101 arrested the cell cycle at the G1 phase. Moreover, the percentage of 5-ethynyl-20-deoxyuridine (EdU) incorporation was reduced from 50% of control cells to 19% of miR-101 transfected cells (Figure 2C). Transfection of miR-101 into 4T1-luc2-M cells induced apoptosis by increasing nucleosomal fragmentation and caspase-3 activity (Figures 2D and 2E). By contrast, as-miR-101-mediated knockdown of miR-101 in 4T1-luc2-NM cells, which have very low metastatic potential and high endogenous miR-101 levels, enhanced cell proliferation significantly, but did not promote apoptosis (data not shown). These results demonstrate that miR-101 inhibits the proliferation and induces apoptosis of BrC cells *in vitro*.

The mobility of 4T1-luc2-M cells in wound-healing assays was reduced after transfection with the miR-101 mimic (Figure 2F). Similarly, in Matrigel invasion assays, miR-101 reduced the invasion of 4T1-luc2-M cells (Figure 2G). These results suggest that miR-101 inhibits the migration and invasion of BrC cells *in vitro*.

### CXCR7 is a direct target of miR-101 in BrC

**Figure 3:**
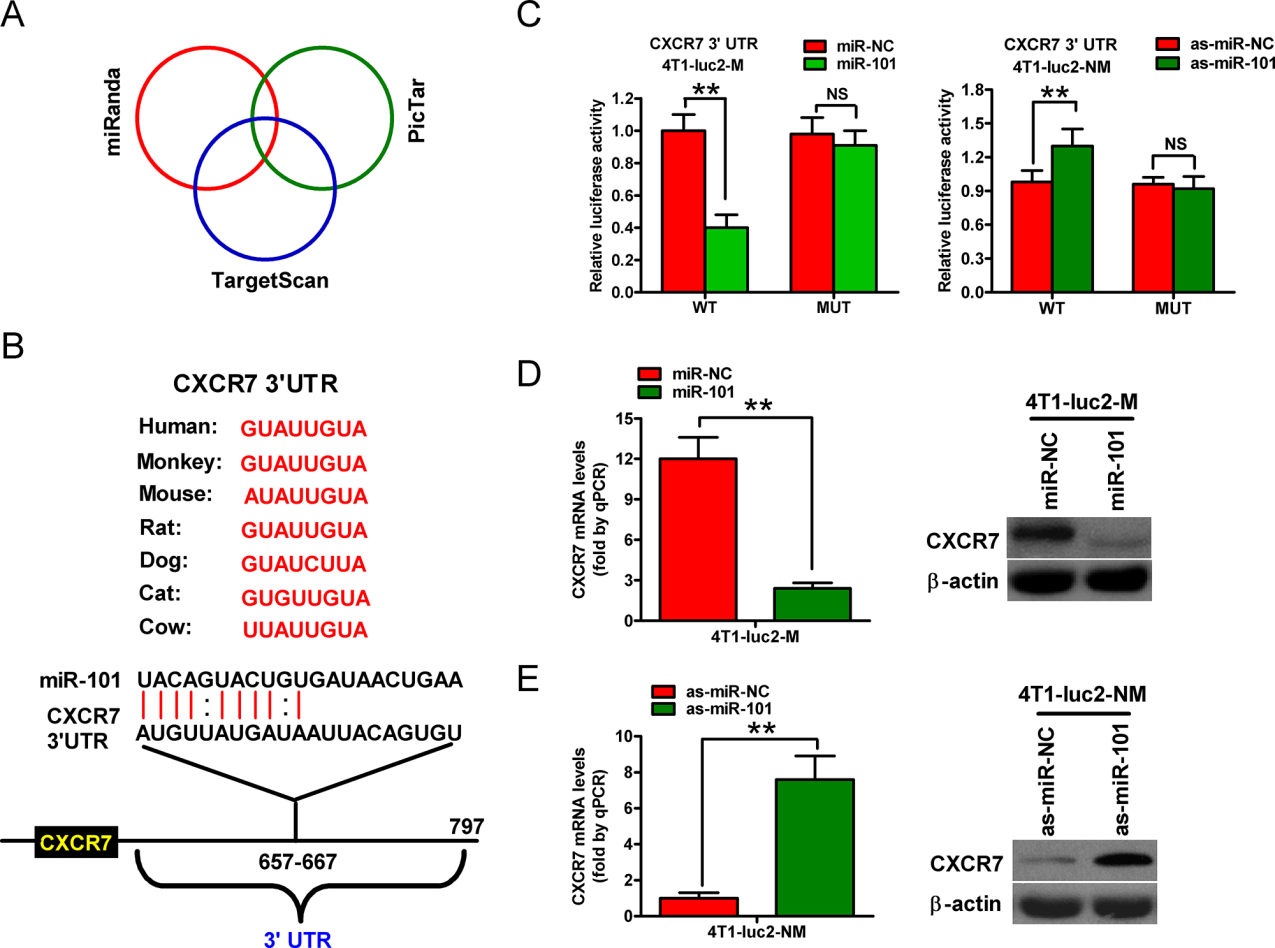
CXCR7 is a direct target of miR-101 **A.** The method used to predict the potential targets of miR-101 by integrating the results of three algorithms (TargetScan, PicTar, and miRanda). **B.** The predicted binding sites of miR-101 in the 3′-UTR of *CXCR7* from various species. **C.** The results of luciferase reporter assays performed 24 h after co-transfection of 4T1-luc2-M or 4T1-luc2-NM cells with a pGL3 construct containing the WT or MUT *CXCR7* 3′-UTR region, and miR-101 or as-miR-101 mimics, respectively. Data were normalized to those from cells co-transfected with pGL3 and miR-NC (4T1-luc2-M cells) or as-miR-NC (4T1-luc2-NM cells). **D.** qRT-PCR and western blot analyses of CXRC7 expression in 4T1-luc2-M cells that were transfected with the miR-101 or miR-NC mimics. **E.** qRT-PCR and western blot analyses of CXRC7 expression in 4T1-luc2-NM cells that were transfected with as-miR-101 or as-miR-NC. (D, E) The *CXCR7* mRNA level was normalized to that of *GAPDH*, and the expression level of β-actin was used as a control for western blotting. Representative western blots are shown. (C–E) Data are represented as the mean ± SD of three replicates. **P < 0.01.

Next, we searched for candidate target genes of miR-101 using the publicly available databases TargetScan, PicTar, and miRanda (Figure 3A). A complementary miR-101 sequence was identified in the 3′-UTR of the *CXCR7* mRNA (Figure 3B); therefore, this gene was selected for further analysis. Dual reporter assays revealed that introduction of miR-101 in 4T1-luc2-M cells suppressed the activity of a luciferase reporter fused to the wild-type (WT) 3′-UTR of *CXCR7*, but did not suppress that of a reporter fused to a mutant (MUT) version of the 3′-UTR (Figure 3C, left). By contrast, downregulation of miR-101 by transfection of 4T1-luc2-NM cells with as-miR-101 increased the activity of a luciferase reporter fused to the WT *CXCR7* 3′-UTR (Figure 3C, right).

The mRNA and protein levels of CXCR7 in 4T1-luc2-M and 4T1-luc2-NM cells were also determined (Supplementary Figure S3). Introduction of miR-101 in 4T1-luc2-M cells reduced CXCR7 expression at the mRNA and protein levels (Figure 3D), whereas knockdown of miR-101 increased these levels in 4T1-luc2-NM cells (Figure 3E). These results suggest that CXCR7 is a direct target of miR-101 in BrC cells.

### CXCR7 is the key mediator of the effects of miR-101 on BrC cells

**Figure 4:**
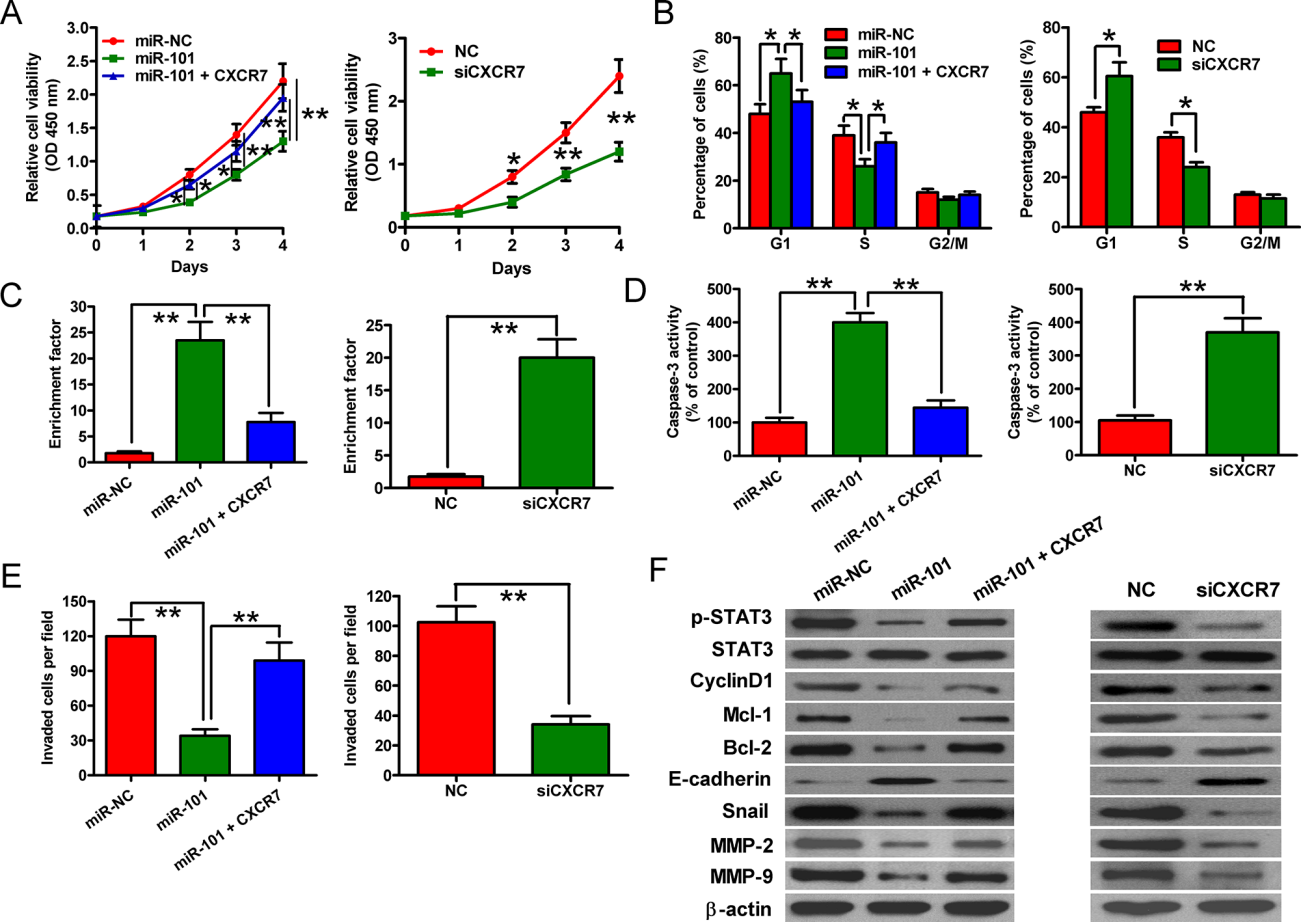
CXCR7 expression and downstream signaling are inhibited by miR-101 in BrC cells **A–E.** In the presence of 100 ng/ml CXCL12, the effects of transfecting 4T1-luc2-M cells with the miR-NC or miR-101 mimic, the miR-101 mimic plus the CXCR7 expression plasmid, siCXCR7, or siNC on cell proliferation (A), cell cycle distribution (B), apoptosis (C, D), and invasive ability (E) were examined. Data are represented as the mean ± SD of three replicates. *P < 0.05 and **P < 0.01. **F.** Western blot analyses of STAT3 and its downstream targets in 4T1-luc2-M cells that were transfected with the miR-NC or miR-101 mimic, the miR-101 mimic plus the CXCR7 expression plasmid, siNC, or siCXCR7 in the presence of 100 ng/ml CXCL12. The expression level of β-actin was used as a loading control. Representative blots are shown.

To confirm that CXCR7 is a functional target of miR-101, 4T1-luc2-M cells were treated with a small interfering RNA (siRNA) against CXCR7 (siCXCR7), a negative control siRNA (si-NC), a negative control miRNA (miR-NC) or miR-101 mimic, or a plasmid expressing CXCR7 in the presence of 100 ng/ml CXC motif chemokine 12 (CXCL12). Exogenous portion of CXCR7 in 4T1-luc2-M cells transfected with CXCR7 plasmid was detected by Western blotting (Supplementary Figure S4). The reduction in cell proliferation caused by ectopic miR-101 was attenuated markedly by co-transfection with the CXCR7 plasmid (Figure 4A, left). Overexpression of CXCR7 also rescued miR-101-induced cell cycle arrest (Figure 4B, left) and apoptosis, as evidenced by decreased nucleosomal fragmentation and caspase-3 activity (Figures 4C and 4D, left). The inhibitory effect of miR-101 on BrC cell invasion was neutralized by CXCR7 overexpression (Figure 4E, left). Furthermore, transfection of 4T1-luc2-M cells with CXCR7-targeted siRNA to knockdown CXCR7 mRNA and protein expression (Supplementary Figure S3) inhibited cell proliferation (Figure 4A, right), induced G1 arrest (Figure 4B, right), increased apoptosis (Figures 4C and 4D, right), and suppressed invasion (Figure 4E, right). These results were consistent with the effects of miR-101 and thus provided further evidence that CXCR7 is a downstream target of miR-101.

To explore the molecular mechanisms by which miR-101 exerts its tumor-suppressive effects, we examined the effects of this miRNA on the activation of STAT3, which is the predominant mediator of CXCR7 signaling, and the protein levels of STAT3 targets, including cyclin D1, Mcl-1, Bcl-2, E-cadherin, Snail, matrix metalloproteinase 2 (MMP2), and MMP9. The levels of phosphorylated STAT3 (p-STAT3) and the STAT3 signaling effectors were reduced in 4T1-luc2-M cells transfected with the miR-101 mimic, whereas co-transfection of the cells with the CXCR7 plasmid reversed these effects (Figure 4F, left). Furthermore, the effects of knockdown of CXCR7 on the expression levels of CXCR7 and its target genes were similar to those induced by ectopic miR-101 (Figure 4F, right). These data indicate that miR-101 inhibits CXCR7-STAT3 signaling in BrC cells.

### Overexpression of miR-101 and depletion of CXCR7 suppress *in vivo* tumor growth, metastasis, and apoptosis of BrC cells

**Figure 5:**
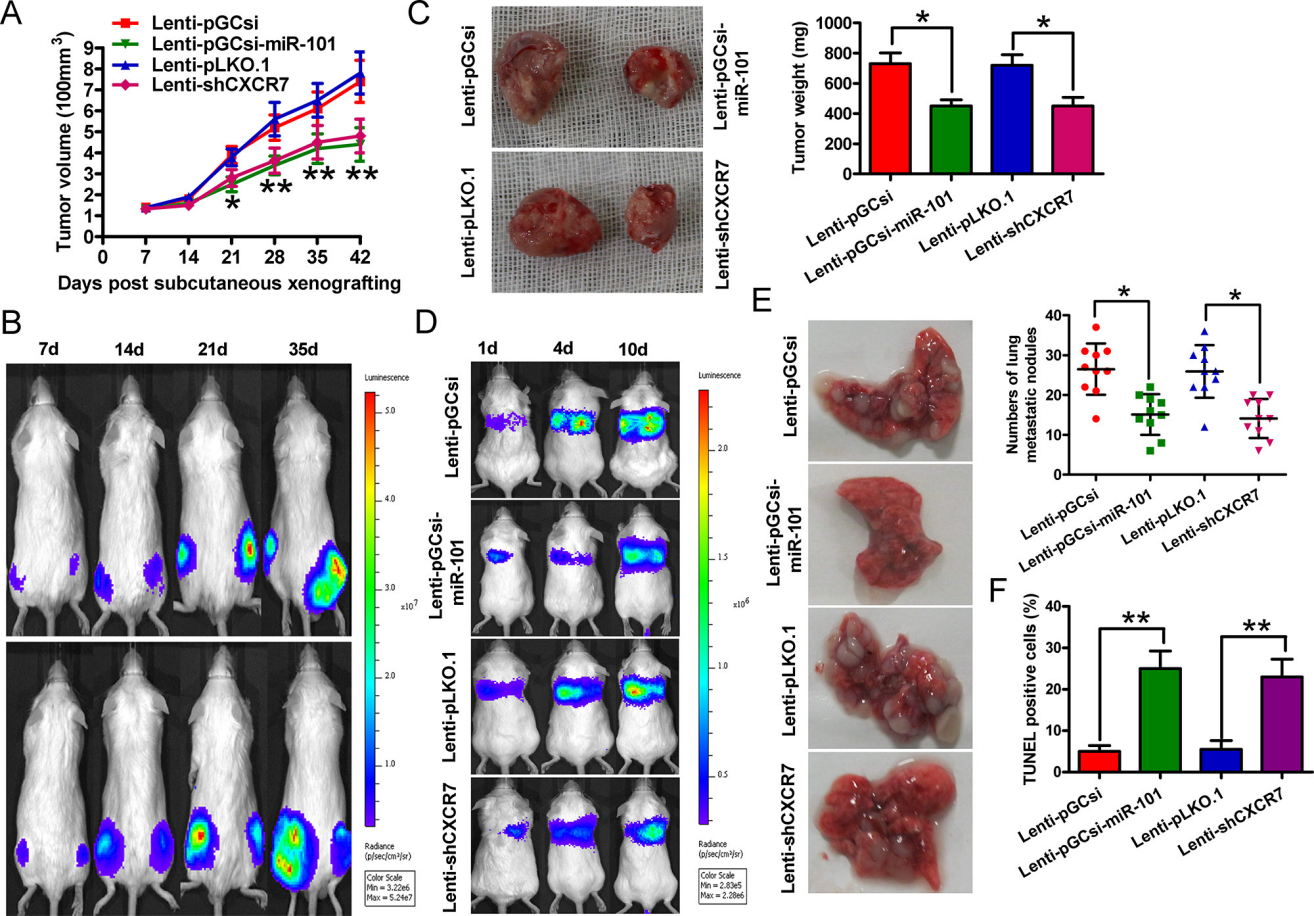
Overexpression of miR-101 or knockdown of CXCR7 suppresses tumor growth and metastasis, and promotes apoptosis of BrC cells *in vivo* Female BALB/c mice (5–7 weeks old) were injected subcutaneously or through the tail vein with 4T1-luc2-M cells that were infected with a control lentivirus (Lenti-pGCsi or Lenti-pLKO.1) or a recombinant lentivirus expressing a miR-101 precursor (Lenti-pGCsi-miR-101) or shCXCR7 (Lenti-shCXCR7) (*n* = 5 mice/group). **A.** Tumor volumes of subcutaneous BrC cell implantation models. **B.** Tumor growth progression in the implanted mice, as determined by *in vivo* luciferase imaging of the xenografts on days 7, 14, 21, and 35 after subcutaneous injection. Upper panel: left hind, Lenti-pGCsi-miR-101; right hind, Lenti-pGCsi. Lower panel: left hind, Lenti-pLKO.1; right hind, Lenti-pLKO.1-shCXCR7. **C.** Representative images of tumors and quantification of tumor weights 6 weeks after subcutaneous xenografting. **D.** Tumor metastasis progression in the implanted mice, as determined by *in vivo* luciferase imaging of lung metastases on days 1, 4, and 10 after tail injection. **E.** Representative images of mouse lungs and quantification of microscopic nodules in the lungs of each group 8 weeks after tail vein injection. **F.** TUNEL assay showing the percentage of apoptotic cells in each group. (B–E) Representative images are shown. (A, C, E, F) Data represent the mean ± SD of three replicates. *P < 0.05 and **P < 0.01.

Subcutaneous injection of 4T1-luc2-M cells stably expressing a miR-101 precursor or a short hairpin RNA specific to CXCR7 (shCXCR7) into BALB/c mice produced tumors within 1 week. The tumor volumes were measured each week, and the mice were sacrificed 6 weeks after tumor cell implantation. As a result, overexpression of miR-101 or knockdown of CXCR7 reduced the *CXCR7* mRNA level and the CXCR7 and p-STAT3 protein levels in BrC tumors (Supplementary Figure S5). The volume and weight of the tumors derived from 4T1-luc2-M cells stably expressing miR-101 were lower than those of the tumors in the control group (Figures 5A–5C). The rate of lung metastasis was also lower in xenograft tumors overexpressing miR-101 than in tumors derived from control cells (Figures 5D and 5E). Furthermore, the number of terminal transferase-mediated dUTP nick end labeling (TUNEL)-positive cells was higher in tumors overexpressing miR-101 than in control tumors (Figure 5F). Similar to the effects of miR-101 overexpression, knockdown of CXCR7 inhibited tumor growth and lung metastasis, and induced BrC apoptosis (Figures 5A–5F). These results suggest that the suppressive effects of miR-101 on *in vivo* BrC tumor growth, lung metastasis, and apoptosis are mediated by reduced CXCR7 expression.

### High CXCR7 and low miR-101 levels correlate with aggressive characteristics of clinical BrCs

**Figure 6:**
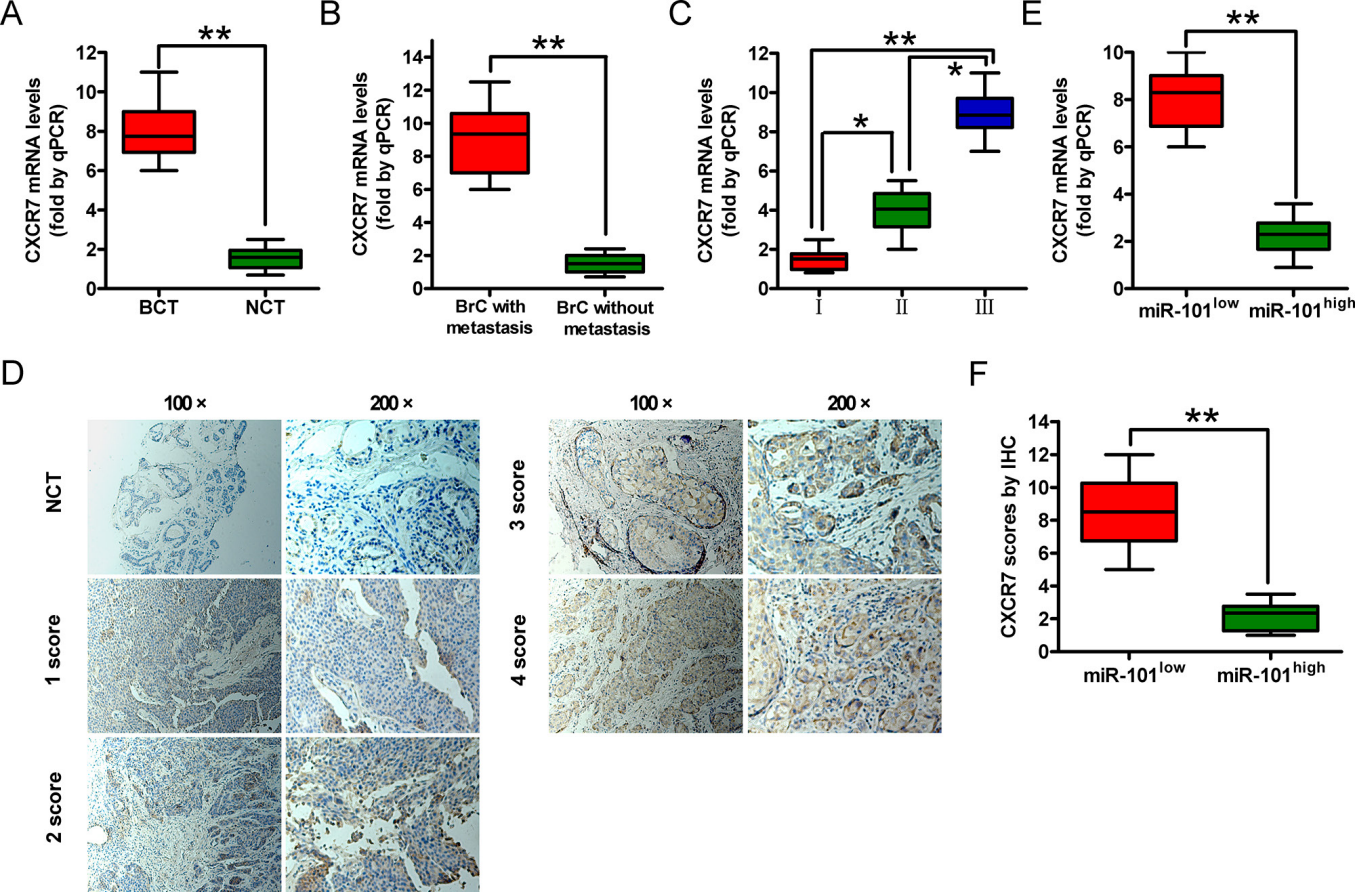
CXCR7 expression in BrC tissues and its correlation with miR-101 expression **A.** A qRT-PCR analysis of *CXCR7* levels in BrC tissues (BCT, *n* = 72) and corresponding non-cancerous tissues (NCT, *n* = 72). **B.** A qRT-PCR analysis of *CXCR7* mRNA levels in BrC tissues with (*n* = 51) or without (*n* = 37) lymphatic metastasis. **C.** A qRT-PCR analysis of *CXCR7* mRNA levels in BrC patients at different clinical stages (WHO I, *n* = 11; WHO II, *n* = 30; and WHO III, *n* = 46). **D.** Representative IHC images of CXCR7 in NCT and BCT with various scores (100× and 200× magnifications). **E.** A qRT-PCR analysis of *CXCR7* mRNA levels in BrC patients with low and high miR-101 levels. **F.** IHC scores of the CXCR7 protein level in patients with low and high miR-101 levels. Scoring was based on the percentage of positive cells with the following staining intensities: less than 5% scored 0, 5–20% scored 1, 20–50% scored 2, 50–75% scored 3, and > 75% scored 4. (A–D, F) Data are represented as the mean ± SD of three replicates. *P < 0.05 and **P < 0.01.

We next examined the clinical relevance of altered miR-101 and CXCR7 expression on BrCs. We found that the levels of the *CXCR7* mRNA were higher in BrC tissues than in non-cancerous tissues (Figure 6A) and correlated with lymphatic metastasis and the histological grade of the tumor (Figures 6B and 6C). We measured CXCR7 protein levels in human BrC (*n* = 248) and matched non-cancerous tissues using immunohistochemical staining. CXCR7 levels were higher in BrC patients with lymphatic metastasis than in those without metastasis (*P* < 0.0001). The CXCR7 protein levels (Figure 6D) and their correlations with the clinicopathological characteristics of BrC are summarized in Supplementary Table S1. High mRNA and protein levels of CXCR7 were positively correlated with the histological grade and lymphatic metastasis in BrC patients. These findings indicate that CXCR7 promotes BrC progression in patients.

We also investigated the relationship between miR-101 expression and the mRNA and protein levels of CXCR7 in BrC patients. The level of miR-101 was inversely correlated with those of the CXCR7 mRNA and protein (Figures 6E and 6F), suggesting that miR-101 inhibits the malignant phenotypes of BrCs by targeting CXCR7.

## DISCUSSION

Despite significant progress in BrC diagnosis and therapy, the dismal 5 year survival rate of BrC patients has not changed substantially [19]. Nevertheless, poor outcomes could be improved through molecular characterization of BrC tumorigenesis and the identification of biomarkers for targeted therapy of select patients. Although previous studies reported that miR-101 represses the malignant transformation and progression of BrC by targeting Stathmin1, EZH2, and Janus kinase 2 [7–9], the underlying mechanisms by which miR-101 inhibits BrC remain unclear. Here, we found that miR-101 expression was decreased markedly in BrC tissues and cell lines. We also demonstrated that restoration of miR-101 levels suppressed BrC cell proliferation and invasion *in vitro*, induced cell cycle arrest, promoted apoptosis, and inhibited tumor growth and metastasis *in vivo* by targeting CXCR7 directly.

The following findings confirmed that CXCR7 is a functional target of miR-101 in BrC: (1) miR-101 expression levels were inversely correlated with those of CXCR7 in BrC tissues and cell lines; (2) miR-101 reduced CXCR7 levels significantly in BrC cells, whereas downregulation of this miRNA had the opposite effect; (3) ectopic miR-101 reduced the activity of a luciferase reporter fused to the WT, but not the MUT 3′-UTR of *CXCR7*; (4) the effects of miR-101 level on the proliferation, apoptosis, and invasion of BrC cells *in vitro* and *in vivo* were accompanied by inverse changes in CXCR7 levels and CXCR7-STAT3 signaling; and (5) overexpression of CXCR7 abrogated the effects induced by miR-101. Overall, these findings indicate that miR-101 is a tumor suppressor and CXCR7 is a downstream mediator of miR-101 function in BrC.

The overexpression of CXCR7 is associated with malignancy [20] because CXCR7 promotes the growth and metastasis of many types of human cancers [11–14]. Wani *et al*. [21] demonstrated that CXCR7 promotes BrC growth by activating STAT3 signaling. In fact, STAT3 is constitutively activated in 35–60% of BrCs [22, 23], and elevated levels of phosphorylated STAT3 are associated with apoptosis resistance, cell cycle progression, and tumor angiogenesis in invasive BrC tissues [24]. STAT3 activation induces the transcription of a number of target genes, including Bcl-2, Mcl-1, cyclin D1, Snail, MMP2, and MMP9 [23, 25–27]. Consistent with these reports, we established here that miR-101 reduced the levels of p-STAT3, Mcl-1, Bcl-2, cyclin D1, Snail, MMP2, and MMP9 in BrC cells significantly, whereas overexpression of CXCR7 counteracts these effects. Similar to overexpression of miR-101, knockdown of CXCR7 inhibited STAT3 phosphorylation and the expression of STAT3 target genes. These results indicate that miR-101 inhibits the CXCR7-STAT3 signaling pathway in BrC cells.

Cyclin D1 is a crucial mediator of G1 to S progression, and its upregulation results in the rapid growth of BrC cells [28]. Thus, downregulation of cyclin D1 could be a mechanism by which miR-101 suppresses cell proliferation and promotes cell cycle arrest at the G1 phase. Bcl-2 and Mcl-1, which play important roles in blocking the apoptosis of BrC cells, are overexpressed in some BrC tissues [29, 30]. Here, we found that miR-101 downregulated Bcl-2 and Mcl-1 significantly by inhibiting the CXCR7-STAT3 pathway, which led to enhanced BrC cell apoptosis. Epithelial-mesenchymal transition (EMT) is a hallmark of metastatic neoplasms, including BrC, and is involved in the concurrent downregulation of epithelial markers such as E-cadherin and α-catenin, and upregulation of mesenchymal markers such as vimentin, β-catenin, and Snail. Inhibition of STAT3 signaling suppresses the EMT of BrC cells by downregulating the expression of Snail, a transcriptional repressor of E-cadherin [26]. Here, ectopic miR-101 impeded the EMT of BrC cells significantly by inhibiting CXCR7-STAT3 signaling. MMPs are a family of enzymes that proteolytically degrade various components of the extracellular matrix. High levels of certain MMPs are closely correlated with the invasive and metastatic potentials of tumors [31]. Specifically, activated STAT3 increases the invasive abilities of BrC cells by initiating the transcription of the *MMP2* and *MMP9* genes, whereas blocking STAT3 activity suppresses MMP2 and MMP9 expression, tumor invasion, and lung metastasis [32, 33]. Therefore, downregulation of MMP2 and MMP9 can be an important mechanism by which miR-101 inhibits BrC invasion and metastasis.

The fundamental role of miR-101 in tumor cell proliferation, apoptosis, and invasion indicates that it could be a potential prognostic predictor and therapeutic target of cancer. In a previous study, low miR-101 levels were defined as an independent predictor of survival in a large cohort of patients with hepatocellular carcinoma [34]. Here, we revealed that miR-101 is an independent prognostic indicator in BrC patients, and that high levels of CXCR7 are positively correlated with the histological grade and lymphatic metastasis of BrC. The expression level of CXCR7 may be used to predict poor clinical outcomes in patients with BrC [21]. In future studies, we will investigate whether the combination of miR- 101 and CXCR7 expression levels serves better as an independent prognostic indicator than miR-101 or CXCR7 alone for the survival of BrC patients.

Our study has limitations in deciphering the role of CXCL12/CXCR7 axis in miR-101-downregulated BrC. Previously, Wani *et al*. [21] demonstrated that CXCL12 enhanced CXCR7-mediated cell migration and ERK and STAT3 signaling compared to the BrC cells without CXCL12 treatment. Herein, we only have illuminated the inhibitory effects of miR-101 on BrC malignancies in the presence of CXCL12. Next, we shall clarify the role of CXCR7 in breast cancer growth and metastasis with or without CXCL12 stimulation.

In conclusion, the results presented here show that miR-101 is downregulated in BrC and is inversely correlated with tumor growth and metastasis. We also demonstrated that miR-101 is an independent prognostic factor in BrC patients. The results of *in vitro* and *in vivo* studies confirmed miR-101 as a novel inhibitor of the growth and invasion of BrC cells by targeting CXCR7. The tumor-suppressive functions of miR-101 were mediated by inhibition of the CXCR7-STAT3 pathway. These data suggest that deregulation of miR-101 promotes tumor development and metastasis, and highlight the potential role of miR-101 in prognostic evaluation and therapeutic application for BrCs.

## MATERIALS AND METHODS

### Ethics statement

All experimental procedures were approved by the Institutional Review Board of the 150th Central Hospital of PLA (China). Written informed consent was obtained for all patient samples. Animal experiments were approved by the Institutional Committee for Animal Research and were performed in conformity with national guidelines for the care and use of laboratory animals.

### Clinical samples

Frozen and paraffin-embedded BrC, and corresponding non-cancerous tissue samples were obtained from Chinese patients who underwent curative resection in the 150th Central Hospital of PLA. None of the patients had received chemotherapy or radiotherapy before surgery. All specimens were confirmed by pathological examinations. Clinical staging was performed according to the American Joint Committee on Cancer Staging Manual. The clinicopathological characteristics of the BrC patients are summarized in Supplementary Table S1.

### Cell culture

Mouse 4T1-luc2 BrC cells stably expressing highly efficient luciferase were obtained from PerkinElmer (Santa Clara, CA, USA). The 4T1-luc2 cell line was characterized by the provider using a gene profiling analysis, and the cells were used no later than 6 months after receipt. The cell line was cultured in Dulbecco's Modified Eagle Medium (DMEM; Sigma, St. Louis, MO, USA) supplemented with 10% fetal bovine serum (FBS; Gibco, BRL, Grand Island, NY, USA). The cells were routinely grown to 80% confluence at 37°C in a humidified atmosphere containing 5% CO_2_, and cells from passages 2–4 were used for experiments.

### Isolation of invasive and non-invasive BrC cell sublines using transwell chambers

Six-well polycarbonate transwell membrane inserts with 8 mm pores (Corning, NY, USA) were used to isolate 4T1-luc2 sublines with different invasive abilities as previously described method [35]. First, cells that were serum-starved for 24 h were suspended in serum-free DMEM to a final density of 5 × 10^5^ cells/ml. A 1 ml cell suspension was seeded into the top chamber, which was coated with 200 mg/ml Matrigel (BD Biosciences, San Jose, CA, USA), and the chamber beneath the polycarbonate membranes was filled with 2.5 ml of DMEM medium supplemented with 20% FBS to create a chemotactic gradient that stimulated penetration of the cells. Following incubation for 24 h at 37°C, the invasive cells on the underside of the membrane and the non-invasive cells on the top of the membrane were harvested aseptically and were expanded for selection. After ten rounds of selection, the subline that failed to invade through the membranes in all selection rounds was designated as 4T1-luc2-NM, and the subline that was able to migrate through the membranes was designated as 4T1-luc2-M.

### Plasmid and lentivirus constructs

The WT 3′-UTR of *CXCR7* and a variant containing mutations in the putative miR-101 binding sites were inserted downstream of the firefly luciferase gene in the pGL3 vector (Promega, Madison, WI, USA). The primers used to amplify the WT and MUT 3′-UTRs are listed in Supplementary Table S2. The pUNOI-mCXCR7 (open reading frame) plasmid was purchased from InvivoGen (Hong Kong, China). All constructs were confirmed by DNA sequencing. An shRNA was designed to target CXCR7, and a scrambled shRNA was used as a control. Paired deoxyribonucleotide oligos encoding the shRNAs were synthesized, annealed, and cloned into the EcoRI and NcoI sites of the pLKO.1 vector (Addgene, Cambridge, MA, USA). HEK293T cells were co-transfected with the constructs described above and the lentivirus packaging plasmids pCMV-VSVG and pCMV-ΔA.9. The virus in a conditioned medium was harvested, filtered, and used for infection of BrC cells. Cells were selected with puromycin (5 μg/ml; Sigma) to generate stable shRNA-expressing clones. For lentiviral expression of miR-101, cDNA strands corresponding to the pre-miR-101 sequence were synthesized and cloned into the AgeI and EcoRI sites of pGCsi-H1-CMV-GFP (GeneChem, Shanghai, China). HEK293T cells were co-transfected with the recombinant or empty vector and the pMD2.G and psPAX2 packaging plasmids. The virus-containing medium was harvested, filtered, and used for infection.

### Cell transfection

The miR-101 mimics, as-miR-101, siCXCR7, and their cognate control RNAs were synthesized by Qiagen (Hilden, Germany), and their sequences are listed in Supplementary Table S3. Transfection was performed using Lipofectamine 2000 reagent (Invitrogen, Carlsbad, CA, USA), according to the manufacturer's instructions. The cells were transfected with 100 nM miR-101 mimics, as-miR-101, siCXCR7, or their cognate controls in 6-well plates. For plasmid transfections, 2 μg of DNA was used.

### Cell stimulation

Cell stimulation was performed as described earlier [21]. In brief, cells were serum starved for 5 hours at 37°C. Serum-starved cells were stimulated with 100 ng/ml CXCL12 and incubated at 37°C for various time periods. At the end of the stimulation, cells were harvested for analysis.

### Quantitative RT-PCR

Total RNA was extracted from fresh tissues and cell lines using TRIzol reagent (Invitrogen), according to the manufacturer's protocol. Reverse transcription was performed using SuperScript™ II Reverse Transcriptase (Invitrogen), and cDNAs were amplified and detected using SYBR Premix Ex Taq™ (TaKaRa, Otsu, Shiga, Japan). To quantify miRNAs, total RNA was reverse-transcribed using the miScript Reverse Transcription Kit (Qiagen) and then amplified using SYBR Premix Ex Taq™ (TaKaRa). The 2^−ΔΔCT^ method was used to determine relative gene expression, and the levels of mRNAs and miRNAs were normalized to those of *GAPDH* and U6, respectively. The primers used for PCR amplification are listed in Supplementary Table S4.

### Western blotting

Proteins were extracted from fresh tissues and cells, separated by sodium dodecyl-sulfate polyacrylamide gel electrophoresis, transferred onto nitrocellulose membranes (Millipore, Bedford, MA, USA), and subjected to immunoblot analyses. Blotting was performed with primary antibodies targeting CXCR7, E-cadherin, Snail (all from Abcam, Cambridge, UK), cyclin D1, MMP2, MMP9 (all from Abnova, Taiwan, China), STAT3, p-STAT3 (Tyr-705), Bcl-2, Mcl-1 (all from Cell Signaling Technology, Danvers, MA, USA), and β-actin (Sigma). The signals were detected using a horseradish-peroxidase-conjugated secondary antibody (Sigma), and the bands were visualized using an enhanced chemiluminescence kit (Santa Cruz, Dallas, TX, USA). Protein bands were quantified using Quantity One software (BioRad, USA).

### Luciferase reporter assay

Cells were co-transfected with reporter constructs, an internal control vector (pGL4.73), and a synthetic miR-101 or as-miR-101 mimic. Forty-eight hours after transfection, luciferase activity was determined using the Dual-Luciferase Reporter Assay System (Promega, Madison, WI, USA) and a luminometer (Glomax 20/20; Promega), and was normalized to the activity of *Renilla* luciferase driven by a constitutively expressed promoter in the phRL vector. Basal promoter activity was measured as the fold-change relative to that observed for the basic pGL3 vector alone.

### Cell proliferation assay

Cell proliferation was measured using Cell Counting Kit-8 (CCK-8; Dojindo Laboratories, Kumamoto, Japan). In accordance with the manufacturer's instructions for CCK-8, harvested cells were seeded into 96-well plates at a density of 1 × 10^3^ cells/well (*n* = 5 for each time point) in a final volume of 100 μl. The cells were cultured for 1, 2, 3, or 4 days after transfection. CCK-8 solution (10 μl) was added to each well, and the absorbance at 450 nm was measured after incubation for 2 h at 37°C to calculate the number of viable cells.

### EdU incorporation assay

Forty-eight hours after transfection with the RNA oligonucleotides, cells in 6-well plates were treated with 50 μM EdU (RiboBio, Guangzhou, China) in culture medium for 2 h, and then fixed with 4% paraformaldehyde for 20 min at room temperature. After washing twice with cold phosphate-buffed saline (PBS), six random fields were selected and photographed under an inverted fluorescent microscope (Carl-Zeiss, Berlin, Germany).

### Cell cycle analysis

Forty-eight hours after transfection with RNA oligonucleotides, the cells were fixed with ice-cold 70% ethanol and treated with 1 mg/ml RNase for 30 min at 37°C. Intracellular DNA was labeled with propidium iodide (50 μg/ml; Sigma) at 4°C for 30 min, and then analyzed using a flow cytometer (Guava Technologies, Carlsbad, CA, USA). The proportions of cells in the G1, S, and G2-M phases were calculated using ModFit software (Verity Software House Inc., Topsham, ME, USA).

### Wound-healing and invasion assay

For the wound-healing assay, 1 × 10^6^ cells were seeded onto 6 cm plates coated with 10 μg/ml type I collagen (Sigma). The cells were grown to 70% confluence, and the monolayer was disrupted with a cell scraper, washed, and incubated in DMEM containing 100 ng/ml CXCL12. Images were taken at 0 and 24 h post-injury using a phase-contrast microscope (Olympus, Tokyo, Japan). The extent of migration into the wound area was evaluated qualitatively using ImageJ software (NIH Image, USA). For the invasion assay, a transwell insert with a diameter of 8 μm (Costar, Dallas, TX, USA) was coated with 200 μl of Matrigel (BD Biosciences, San Jose, CA, USA) at a concentration of 200 μg/ml, and then pre-incubated with DMEM. Cells were seeded into the upper chamber of the transwell (2 × 10^4^ cells/insert), and DMEM containing 100 ng/ml CXCL12 was added to the lower chamber. After incubation at 37°C for 24 h, the cells were fixed in methanol and stained with DAPI or crystal violet (Sigma). Cells that invaded through the pores to the lower surface of the filter were counted under a microscope. Three invasion chambers were used per condition, and the total numbers of cells from the three filters were averaged.

### Nucleosomal fragmentation and caspase-3 activity assays

Forty-eight hours after transfection with the RNA oligonucleotides, cell apoptosis was quantified using a nucleosomal fragmentation kit (Cell Death Detection ELISA PLUS; Roche Applied Science, Indianapolis, IN, USA), according to the manufacturer's protocol. The absorbance values were normalized to those of control cells to derive a nucleosomal enrichment factor. Caspase-3 activity was determined using the Caspase-3/CPP32 Colorimetric Assay Kit (Biovision, Palo Alto, CA, USA). Briefly, 1 × 10^6^ cells were lysed, and the supernatant was collected after centrifugation at 10,000 × *g*. Protein (50 μl; 150 μg) was added to 50 μl of 2× reaction buffer and 5 μl of N-acetyl-Asp-Glu-Val-Asp-pNA substrate (final concentration, 200 μM). After incubation at room temperature for 1–2 h, N-scetyl-Asp-Glu-Val-Asp-pNA cleavage was monitored using a microplate reader (Bio-Tek instruments Inc., Winooski, VT, USA). The absorbance of each well at 405 nm was detected to monitor enzyme-catalyzed release of pNA.

### TUNEL assay

A TUNEL assay was used to detect DNA strand breaks *in situ*, according to the manufacturer's protocol (Roche, Mannheim, BW, Germany). In brief, tissue specimens were fixed with 10% formalin overnight, embedded with paraffin, non-serially sectioned (4 μm), and mounted onto poly-lysine-coated slides. After deparaffinization in xylene and rehydration in a graded series of ethanol solutions, the sections were rinsed with PBS and incubated with FITC-labeled terminal deoxynucleotidyl transferase nucleotide mix at 37°C for 60 min. Subsequently, the sections were rinsed twice in PBS and counterstained with 10 mg/ml DAPI. TUNEL-positive cells were imaged and mounted using a fluorescent microscope (Carl-Zeiss), and were ultimately expressed as a percentage of the total cells determined by DAPI staining.

### Immunohistochemistry and scoring

The streptavidin-peroxidase method was used to detect CXCR7 expression in 248 BrC and non-cancerous tissue samples by immunohistochemistry (IHC). The tissues were sectioned, treated with 3% H_2_O_2_, and then incubated in 5% goat antiserum. Non-serial tissue sections were incubated with a primary antibody against CXCR7 (1:100; Abnova) overnight, and then with a biotin-labeled secondary antibody. Streptavidin-peroxidase complex was added, and the sections were stained with 3,3′-diaminobenzidine (Maixin Biotech., Fuzhou, China) prior to microscopy analyses. CXCR7 immunostaining was scored by two experienced pathologists. Scoring was based on the percentages of positive cells with the following staining intensities: less than 5% scored “–”, 5–20% scored “+”, 20–50% scored “++”, 50–75% scored “+++”, and more than 75% scored “++++”.

### *In vivo* tumor growth, metastasis, and apoptosis assays

The hind flanks of female 5–7-week-old BALB/c mice (Institute of Zoology, Chinese Academy of Sciences) were injected subcutaneously with 1 × 10^6^ 4T1-luc2-M cells that were infected with a control lentivirus or a recombinant lentivirus expressing a miR-101 precursor or shCXCR7 (*n* = 5 mice/group). The tumor volume was monitored and calculated as follows: tumor volume = width^2^ × length/2. All mice were sacrificed by euthanasia at 6 weeks post-inoculation, and the tumors were removed and weighed. The infected 4T1-luc2-M cells were also used for *in vivo* metastasis assays (*n* = 5 mice/group). Female 5–7-week-old BALB/c mice were injected with 1 × 10^6^ cells through the tail vein. The mice were monitored for general health status and evidence of morbidity related to the primary tumor or metastasis, and then sacrificed by euthanasia at 8 weeks post-inoculation. Anatomized mice were examined for metastasis in the lung. Lungs with visible tumor colonies were fixed and embedded in paraffin, and three non-sequential sections per animal were obtained. The sections were stained with hematoxylin and eosin (Maixin Biotech.) and analyzed for the presence of metastasis by light microscopy. The total numbers of metastases per lung section were counted and averaged. For apoptosis assays, the tissue sections were stained using TUNEL kits. TUNEL-positive cells were examined under a fluorescent microscope.

### Bioluminescence imaging and quantification

Female 5–7-week-old BALB/c mice received 1 × 10^6^ 4T1-luc2-M cells (in 100 μl of PBS) that were infected with a control lentivirus or a recombinant lentivirus expressing a miR-101 precursor or shCXCR7 via subcutaneous injection or through the tail vein. Tumor growth and metastasis progression was measured by *in vivo* luciferase imaging of the xenografts at days 7, 14, 21, and 35, and the lung metastases at days 1, 4, and 10 after treatment. The *in vivo* luciferase imaging was performed by intraperitoneal injection of the mice with D-luciferin (Promega, Madison, WI, USA) at a dose of 150 mg/kg per mouse. The mice were anesthetized, and images were acquired using the Xenogen IVIS imaging system. The signals in defined regions of interest were quantified as photon flux (photons/s/cm^2^) using Living Image software (Xenogen Corporation, Berkeley, CA, USA).

### Statistical analysis

Statistical analyses were performed using SPSS version 16.0 software (SPSS Inc., Chicago, IL, USA). Student's *t*-tests were used to analyze the results expressed as the mean ± standard derivation (SD), Chi-squared tests were used to evaluate frequencies, and Fisher's exact test was used for comparisons of qualitative variables. A log-rank test was used for the survival analysis. *P* < 0.05 was considered significant.

## SUPPLEMENTARY FIGURES AND TABLE



## Abbreviations

as-miR-101: anti-miR-101
BrC: breast cancer
CCK-8: cell counting kit-8
CXCL12: CXC motif chemokine 12
CXCR7: CX chemokine receptor 7
DMEM: Dulbecco's Modified Eagle Medium
EdU: 5-ethynyl-20-deoxyuridine
EMT: epithelial-mesenchymal transition
EZH2: enhancer of zeste homolog 2
IHC: immunohistochemistry
Mcl-1: myeloid cell leukemia-1
miR-101: microRNA-101
MMP: matrix metalloproteinase
MUT: mutant
qRT-PCR: quantitative reverse transcription-polymerase chain reaction
SD: standard derivation
STAT3: signal transducer and activator of transcription 3
shRNA: short hairpin RNA
siRNA: small interfering RNA
TUNEL: terminal transferase-mediated dUTP nick end labeling
UTR: untranslated region
WT: wild-type
