# Supplementary material for: MiRNA-101 inhibits breast cancer growth and metastasis by targeting CX chemokine receptor 7
Source: Oncotarget. 2015 Sep 1;6(31):30818–30. doi: 10.18632/oncotarget.5067 (PMC4741570; doi:10.18632/oncotarget.5067)
Supplement: Supplementary file 1 [file oncotarget-06-30818-s001.pdf]

SUPPLEMENTARY FIGURES AND TABLES

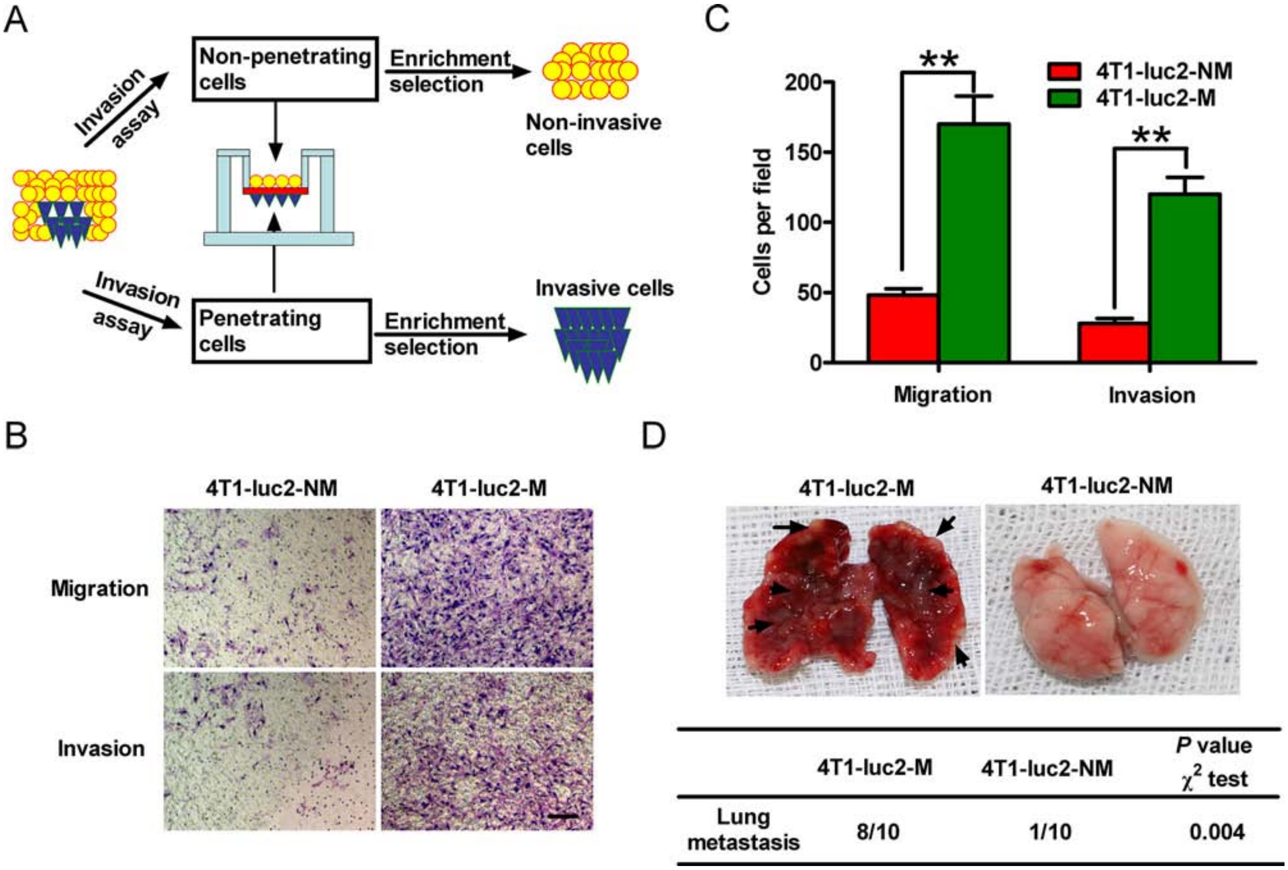

Supplementary Figure S1: Metastatic characteristics of BrC cell sublines. A. Overview of the establishment of invasive and non-invasive sublines derived from the mouse 4T1-luc2 BrC cell line. **B, C.** *In vitro* migration and invasion activities of the BrC cell sublines. The activities were measured *in vitro* using transwell chambers, as described in the Materials and Methods. (B) Representative fields of invasive cells on the membrane. (C) Data represent the mean ± SD of three replicates. $^{**}P < 0.01$. Scale bar = 10 µm. **D.** Metastatic potentials of the BrC cell sublines *in vivo* ($n = 10$). The formation and incidences of lung metastasis in mice that received intravenous tail injections of the cell sublines (4T1-luc2-NM and 4T1-luc2-M). Representative images of mouse lungs are shown.

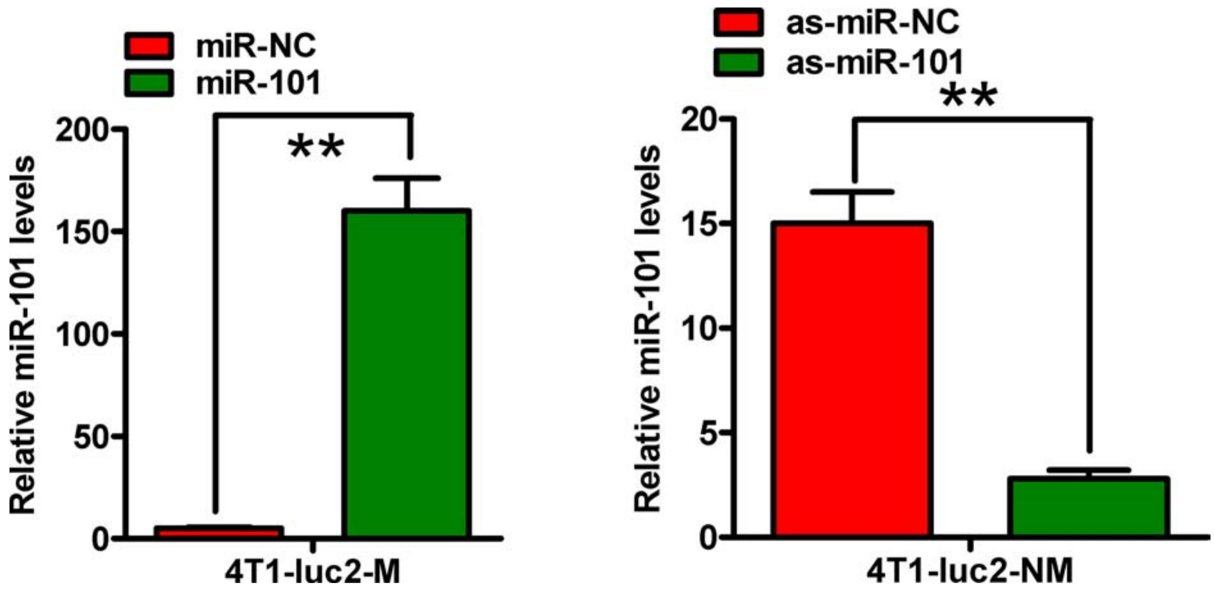

Supplementary Figure S2: MiR-101 expression in 4T1-luc2-M and 4T1-luc2-NM cell lines subjected to different treatments. Quantitative RT-PCR assays of 4T1-luc2-M cells transfected with miR-101 mimics or a negative control mimic (miR-NC), and 4T1-luc2-NM cells transfected with as-miR-101 or as-miR-NC. The expression level of miR-101 was normalized to that of U6 RNA. Data represent the mean ± SD of three replicates. ***P* < 0.01.

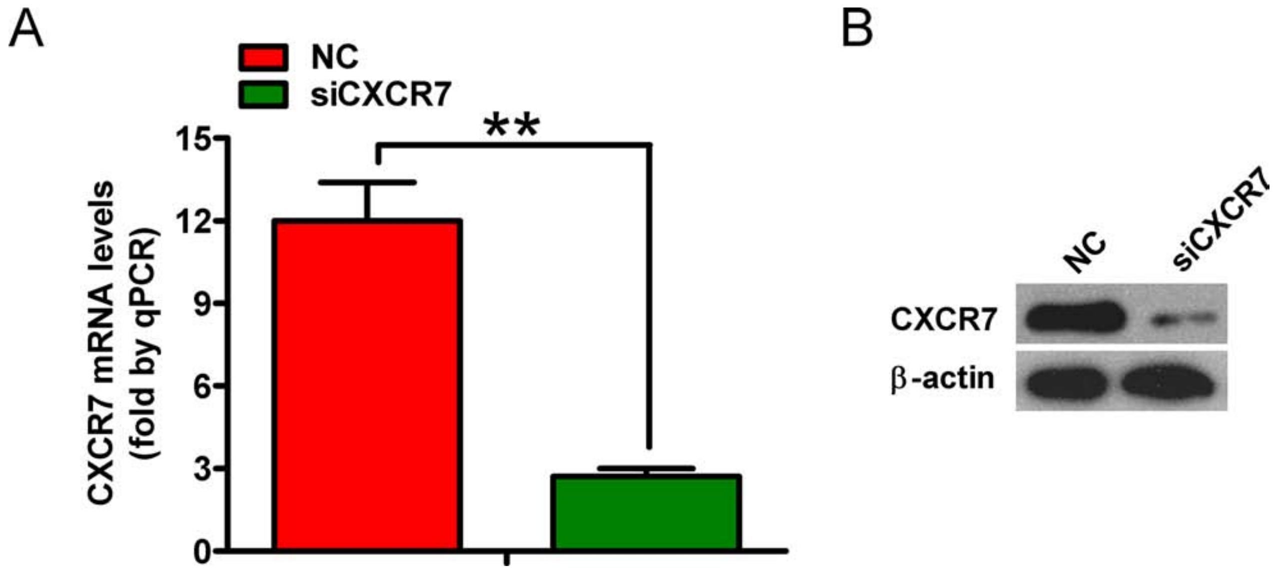

Supplementary Figure S3: The knockdown efficiency of siCXCR7. A, B. Quantitative RT-PCR (A) and western blot (B) analyses of CXCR7 expression in 4T1-luc2-M cells transfected with 100 nM siCXCR7 or the corresponding siNC control. The *CXCR7* mRNA expression level was normalized to that of *GAPDH*, and the expression level of β-actin was used as a control for western blotting. (A) Data are represented as the mean ± SD of three replicates. ***P* < 0.01. (B) A representative western blot is shown.

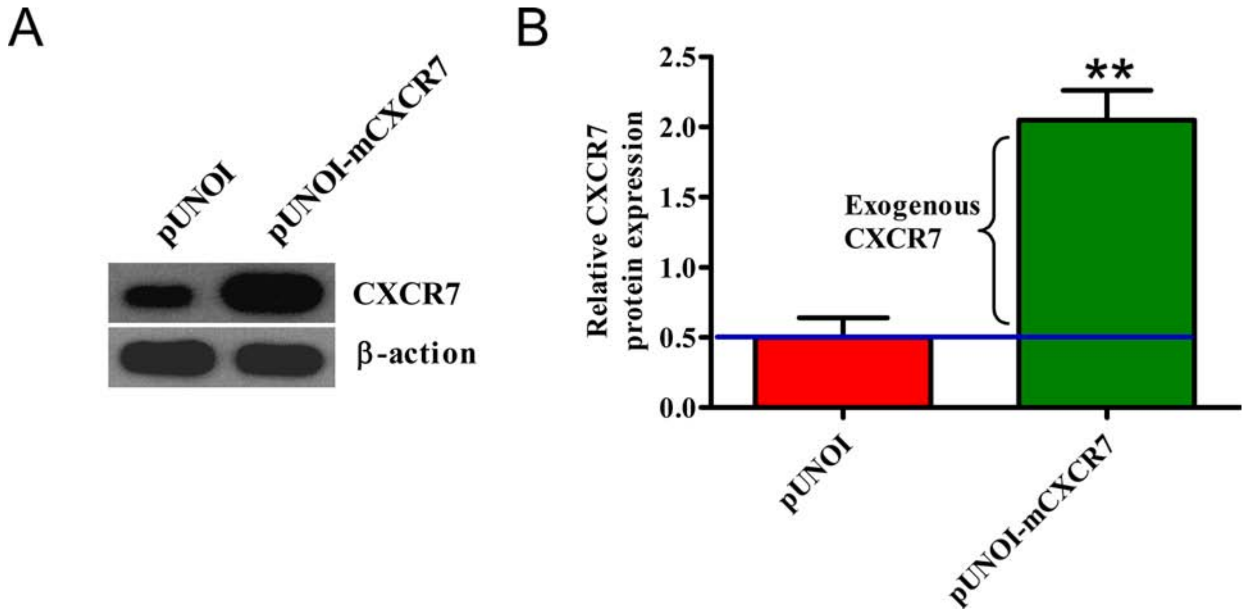

Supplementary Figure S4: Exogenous CXCR7 protein expression in 4T1-luc2-M cells transfected with a CXCR7 plasmid. A. Western blot analyses of CXCR7 expression in 4T1-luc2-M cells transfected with pUNOI or pUNOI-mCXCR7 plasmid. **B.** The relative CXCR7 protein expression in the above groups. Exogenous CXCR7 protein expression was indicated. The CXCR7 protein expression level was normalized to that of β-actin. (A) A representative western blot is shown. (B) Data are represented as the mean ± SD of three replicates. $^{**}P < 0.01$.

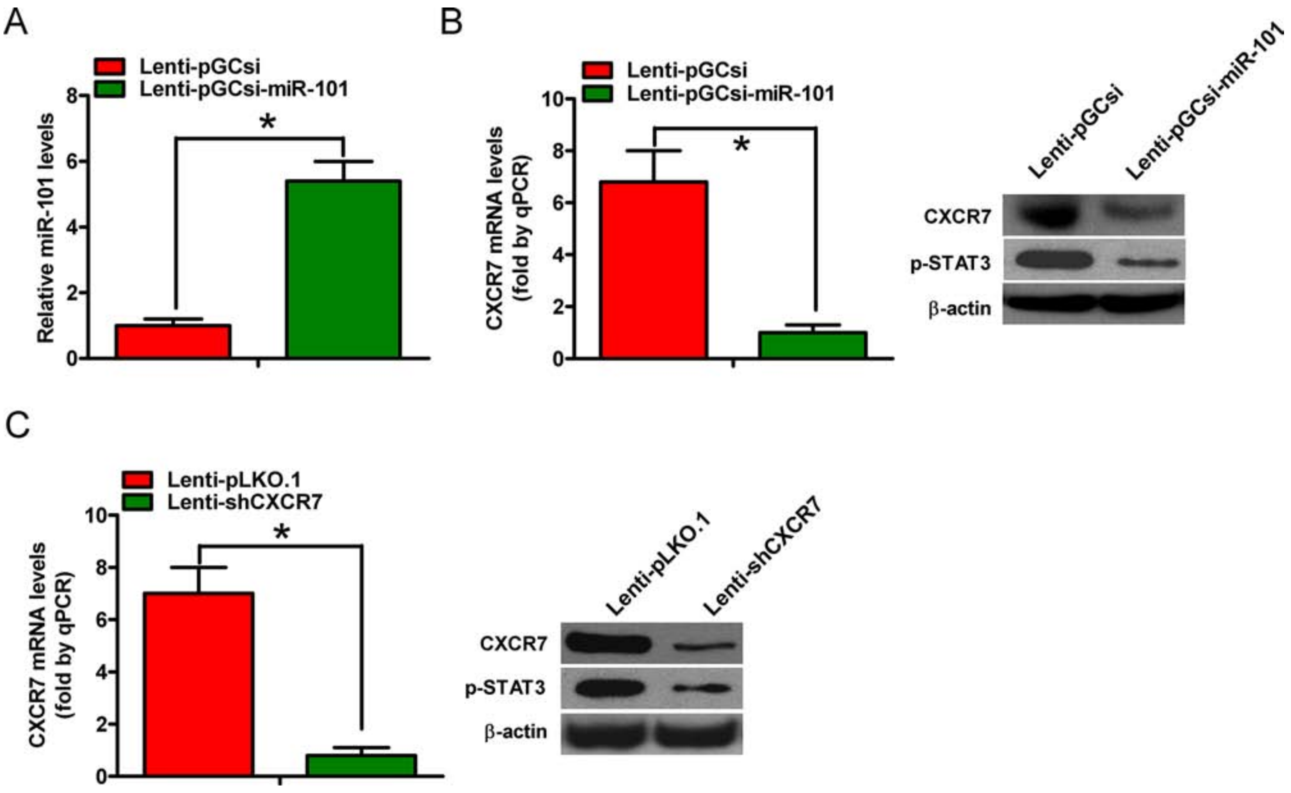

Supplementary Figure S5: Overexpression of miR-101 and knockdown of CXCR7 in xenograft tumors. Female BALB/c mice (5–7 weeks old) were injected subcutaneously with 4T1-luc2-M cells that were infected with a control lentivirus (Lenti-pGCsi or Lenti-pLKO.1) or a recombinant lentivirus expressing a miR-101 precursor (Lenti-pGCsi-miR-101) or shCXCR7 (Lenti-shCXCR7) ($n = 5$ mice/group). The tumors were removed 6 weeks after subcutaneous xenografting. **A.** A qRT-PCR analysis of miR-101 expression in xenograft tumors derived from 4T1-luc2-M cells that were infected with Lenti-pGCsi or Lenti-pGCsi-miR-101. The expression level of miR-101 was normalized to that of U6 RNA. **B.** Western blot and qRT-PCR analyses of CXCR7 protein and mRNA levels in xenograft tumors derived from 4T1-luc2-M cells that were infected with Lenti-pGCsi or Lenti-pGCsi-miR-101. **C.** Western blot and qRT-PCR analyses of CXCR7, and p-STAT3 protein and mRNA levels in xenograft tumors derived from 4T1-luc2-M cells that were infected with Lenti-pLKO.1 or Lenti-shCXCR7. (A–C) Data represent the mean ± SD of three replicates. *$P < 0.05$. (B, C) The *CXCR7* mRNA expression level was normalized to that of *GAPDH*, and the expression level of β-actin was used as a control for western blotting. Representative western blots are shown.

Supplementary Table S1. Correlations between CXCR7 protein levels and the clinicopathological characteristics of BrC patients

Clinical pathology	CXCR7 levels (IHC)			*N*	*P*-value
	− or +	++	+++or ++++		
Age (years):					
≤63	27	37	70	134	0.0894
>63	32	29	53	114	
Carcinoma:					
Primary	61	63	124	248	<0.0001*
Adjacent	127	118	3	248	
Clinical stage:					
I	16	12	14	42	0.0003*
II	13	15	50	78	
III	32	37	59	128	
Lymph node status:					
Metastasis	23	41	83	147	<0.0001*
No metastasis	38	26	37	101	

The *P*-values were calculated using chi-square tests of CXCR7 levels in the different subgroups.
[*] $P < 0.05$.

Supplementary Table S2. The primers used to generate the WT and MUT *CXCR7* 3′-UTRs

Gene	Primer sequences (5′ to 3′)	
CXCR7 3′-UTR(WT)	Forward	GAATTCGACGGGTTTACTTGTTTT
	Reverse	CTGCAGGGAAACAAAAATCTTTAT
CXCR7 3′-UTR (MUT)	Forward	CACCAATAGTGAGAAATATTTCACTTAAAATTTAC
	Reverse	GTATTTAAATTTTAAGTGAAATATTTCTCACTATTG

Supplementary Table S3. The siRNAs and miRNA mimics/inhibitor used in the study

Gene	Sequences (5′ to 3′)	
siRNA CXCR7	Sense	GGAGAGCGUGUAGAGCAGGTT
	Antisense	CCUGCUCUACACGCUCUCCTT
Negative control siRNA	Sense	UUCUCCGAACGUGUCACGUTT
	Antisense	ACGUGACACGUUCGGAGAATT
miR-101 mimic	Syn-mmu-miR-101 miScript miRNA Mimic	
miR-NC	AllStars Neg. Control siRNA	
as-miR-101	Anti-mmu-miR-101 miScript miRNA Inhibitor	
as-miR-NC	miScript Inhibitor Neg. Control	

Supplementary Table S4. The primers used for qRT-PCR analyses

Gene	Primer sequences (5′ to 3′)	
miR-101	Forward	CTACAGTACTGTGATAACTGAA
	Reverse	Universal Primer (QIAGEN)
U6	Forward	RNU6B_2 miScript Primer (QIAGEN)
	Reverse	Universal Primer (QIAGEN)
CXCR7	Forward	AGCCTGGCAACTACTCTGACA
	Reverse	GAAGCACGTTCTTGTTAGGCA
GAPDH	Forward	AGGTCGGTGTGAACGGATTTG
	Reverse	TGTAGACCATGTAGTTGAGGTCA
